# Analysis of the *Tomato spotted wilt virus* Ambisense S RNA-Encoded Hairpin Structure in Translation

**DOI:** 10.1371/journal.pone.0031013

**Published:** 2012-02-21

**Authors:** Christina Geerts-Dimitriadou, Yun-Yueh Lu, Corinne Geertsema, Rob Goldbach, Richard Kormelink

**Affiliations:** Laboratory of Virology, Wageningen University, Wageningen, The Netherlands; University of Kansas Medical Center, United States of America

## Abstract

**Background:**

The intergenic region (IR) of ambisense RNA segments from animal- and plant-infecting (-)RNA viruses functions as a bidirectional transcription terminator. The IR sequence of the *Tomato spotted wilt virus* (TSWV) ambisense S RNA contains stretches that are highly rich in A-residues and U-residues and is predicted to fold into a stable hairpin structure. The presence of this hairpin structure sequence in the 3′ untranslated region (UTR) of TSWV mRNAs implies a possible role in translation.

**Methodology/Principal Findings:**

To analyse the role of the predicted hairpin structure in translation, various *Renilla* luciferase constructs containing modified 3′ and/or 5′ UTR sequences of the TSWV S RNA encoded nucleocapsid (N) gene were analyzed for expression. While good luciferase expression levels were obtained from constructs containing the 5′ UTR and the 3′ UTR, luciferase expression was lost when the hairpin structure sequence was removed from the 3′ UTR. Constructs that only lacked the 5′ UTR, still rendered good expression levels. When in addition the entire 3′ UTR was exchanged for that of the S RNA encoded non-structural (NSs) gene transcript, containing the complementary hairpin folding sequence, the loss of luciferase expression could only be recovered by providing the 5′ UTR sequence of the NSs transcript. Luciferase activity remained unaltered when the hairpin structure sequence was swapped for the analogous one from *Tomato yellow ring virus*, another distinct tospovirus. The addition of N and NSs proteins further increased luciferase expression levels from hairpin structure containing constructs.

**Conclusions/Significance:**

The results suggest a role for the predicted hairpin structure in translation in concert with the viral N and NSs proteins. The presence of stretches highly rich in A-residues does not rule out a concerted action with a poly(A)-tail-binding protein. A common transcription termination and translation strategy for plant- and animal-infecting ambisense RNA viruses is being discussed.

## Introduction

Ambisense genomic RNA segments are quite unique and limited to a number of segmented, negative-strand (-)RNA viruses within the families *Arenaviridae* and *Bunyaviridae* (genus *Phlebovirus* and *Tospovirus*) and the floating genus *Tenuivirus*, and include plant- and animal-infecting viruses [1]. RNA segments with an ambisense gene arrangement contain two non-overlapping genes on opposite strands that are separated by a large intergenic region (IR).

Genes encoded by ambisense RNA segments become expressed by the synthesis of a subgenomic messenger RNA (mRNA) and is initiated with cap-snatching, a process during which the viral RNA polymerase cleaves a capped RNA leader from host cellular mRNAs to use these as primers for transcription on the viral genome. As a result of this, viral transcripts contain a 5′ cap-structure and a non-viral, heterogeneous RNA leader sequence that ranges from a few nucleotides up to 25 nucleotides in size [1]. Studies on the plant-infecting bunyavirus *Tomato spotted wilt virus* (TSWV) and orthomyxovirus *Influenza A* indicate that selection and cleavage of host cellular mRNA leaders involves similar criteria for all segmented (-)RNA viruses [2]–[7].

Transcripts from arenaviruses, bunyaviruses and tenuiviruses all lack a poly(A)-tail like common eukaryotic mRNAs. The 3′ ends of the ambisense-encoded subgenomic viral mRNAs map to the IR, which acts as a (bidirectional) transcription terminator for both encoded genes [8]–[13]. However, viral RNA elements that control transcriptional termination are still largely unknown. Only for arenavirus IR sequences are predicted to fold into single or double stem-loop structures which have been demonstrated to be essential for transcription termination, possibly in the same manner as prokaryotic transcription termination occurs [10], [14]–[16]. The IR sequences of bunyavirus ambisense RNA segments are more diverse in composition. For some (plant- and animal-infecting) bunyaviruses, the IR contains stretches of highly A- and U-rich sequences that enable the formation of a stable hairpin structure [8], [11], [17], [18], while those of others contain G- or C-rich sequences and additionally some conserved sequence motifs [19]. For the Uukuniemi phlebovirus, the IR sequence has been shown to enhance reporter expression in a minireplicon system, which has been explained as a result of efficient transcription termination [20]. The IR sequences of tenuivirus ambisense RNA segments often contain A-rich and/or U-rich sequences but their role in transcription termination has never been further analysed [1].

TSWV is the representative of the plant-infecting tospoviruses within the family *Bunyaviridae* [21] and ranks among the top ten of economically most important plant viruses worldwide [22]–[24]. The TSWV genome comprises three single-stranded RNA segments, denoted small (S), medium (M) and large (L), that distinguishes it from the other, animal-infecting members of the *Bunyaviridae* as two (M and S) out of its three genomic segments contain an ambisense gene arrangement [17], [18]. The S RNA segment contains two non-overlapping open reading frames (ORFs) on opposite strands, coding for the nucleocapsid (N) and non-structural (NSs) protein respectively. The NSs has been shown to be involved in suppression of gene silencing [25], [26]. The N protein tightly associates to the genomic RNA and together with small amounts of the viral RNA-dependent RNA polymerase (RdRP) form transcriptionally active ribonucleoproteins (RNPs), the templates for RNA synthesis (replication and transcription) by the RdRP [24]. TSWV N and NSs genes are separated by a large IR, that contains stretches of highly A- and U-rich sequences which are predicted to fold into a stable hairpin structure [17]. The 3′ ends of the N and NSs transcripts have been mapped within the IR and revealed the presence of the entire hairpin structure encoding sequence [13].

Eukaryotic mRNAs posses a 5′ cap structure and a 3′ poly(A) tail that are involved in bridging the 3′ and 5′ ends of the mRNA [27], [28]. This circularisation supports efficient translation of mRNA, presumably by facilitating recycling of the ribosomal subunits from the 3′ end back to the 5′ end. While bunyavirus mRNAs lack a poly(A) tail, it is not unlikely that such role is assigned to a structural sequence element within the 3′ untranslated region (UTR) that functionally acts as an equivalence.

To test whether the 3′ hairpin structure in TSWV S RNA-derived transcripts plays such role and enhances translation efficiency, various N gene-based constructs were made and tested in BHK-21 animal cells for translation efficiency. These constructs differed in their 3′ termini, i.e. with mutations in the sequence of the predicted hairpin structure. For quantification purposes, the viral N gene was swapped for the *Renilla* (REN) luciferase gene. Results from this analysis are shown and suggest a role of the TSWV hairpin structure in translation, during which the 5′ UTR may act in concert with the hairpin structure.

## Results

### TSWV S segment 3′ UTR is required for translation

**Figure 1:**
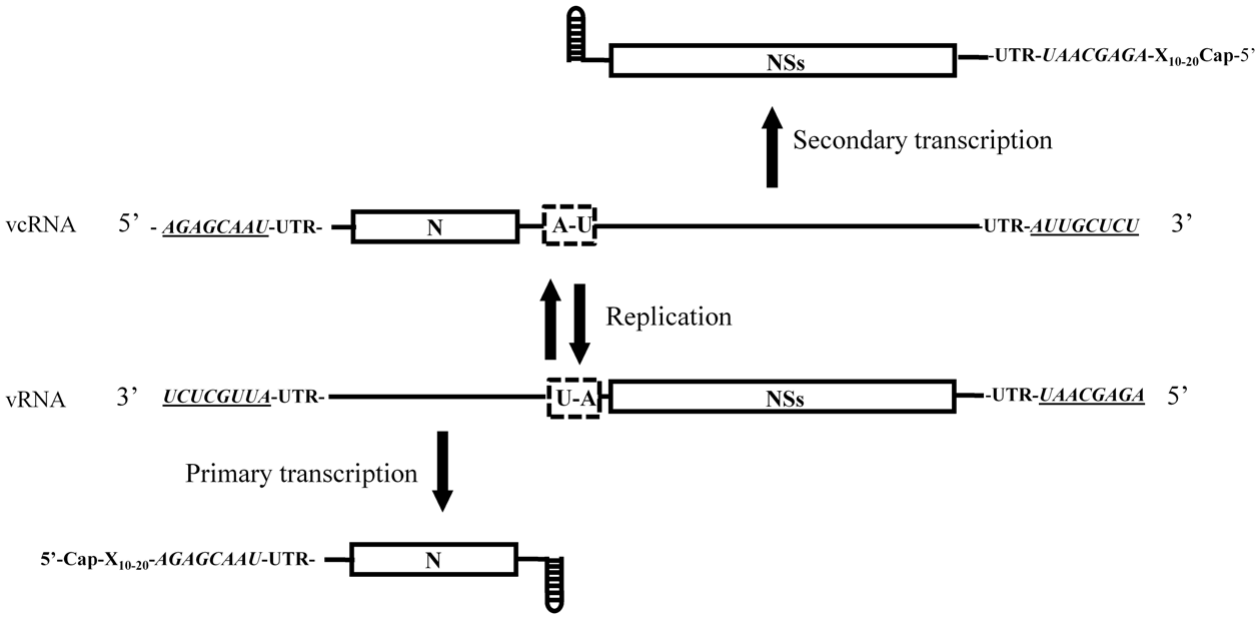
Structural features within the S RNA segment.

**Figure 2:**
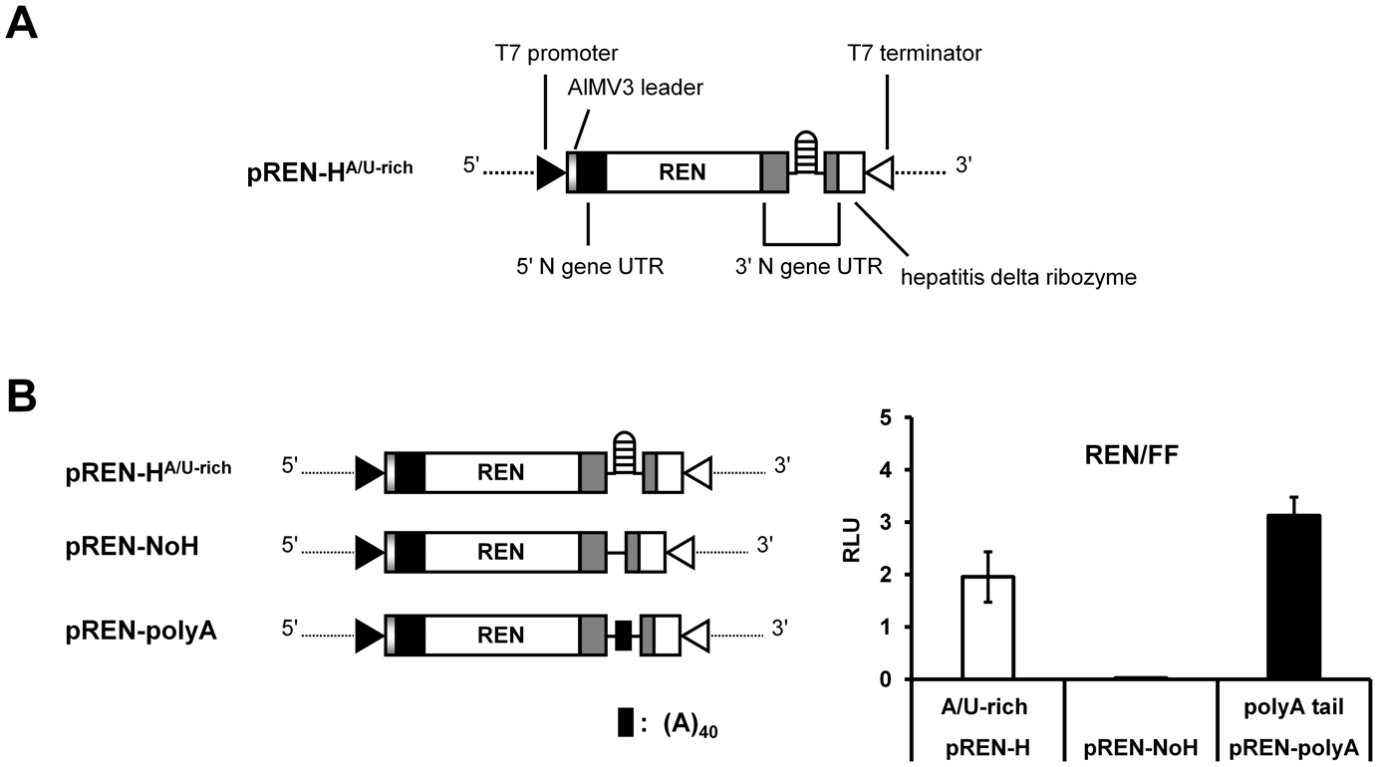
Analysis of the hairpin structure sequence in translation. Schematic presentation of TSWV-N (REN) and derived templates with modifications at the 3′ UTR (A and B). (C) Luciferase activity monitored from REN constructs transfected to BHK-21 cells. Cells were infected with vv-T7 and subsequently co-transfected with 100 ng of the indicated REN constructs and 0.5 ng of the FF luciferase expression plasmid (pIRES-FF) as internal control. The relative luciferase expression (REN/FF) was measured after 23 h post transfection. Error bars indicate standard deviations from the means of three replicate experiments.

To analyse the involvement of the predicted hairpin structure within the IR region of the ambisense TSWV S RNA segment in translation of the S RNA-encoded N and NSs transcripts, translation studies were performed on variants of a model template that reflected authentic viral mRNAs (Fig. 1). To this end, a copy of an N mRNA molecule was made, preceded with a non-viral leader sequence and at its 3′ end flanked with the predicted hairpin structure sequence (see Methods). As a non-viral leader sequence, a copy of the Alfalfa mocaic virus RNA 3 (AlMV3) capped-leader sequence (17 nts) was used, since this sequence were previously observed to *in vivo* prime TSWV genome transcription and render capped, translatable viral transcripts [2], [6], [7]. The entire construct was fused at its 3′ end with a ribozyme sequence to remove all sequences downstream the 3′ end of the viral mRNA transcript after its synthesis, e.g. a poly(A)-tail that could affect or interfere in its translation efficiency, and thus create 3′ ends that would most closely mimic authentic viral transcripts. This strategy has been successfully applied in reverse genetics systems with segmented (-)RNA viruses to generate minireplicons, i.e. RNA molecules with authentic viral genome ends flanking a reporter gene, to study *cis*- and *trans*-acting sequences involved in replication and transcription [29]–[33]. The entire construct was cloned inside a T7 promoter-terminator cassette. For (sensitive) quantification purposes, the viral N gene was next swapped by the REN luciferase gene (Fig. 2A). This construct, marked pREN-H^A/U-rich^, was used for the construction of variants in which the predicted hairpin structure sequence was (partially) deleted, mutated, or exchanged for another tospovirus hairpin sequence and subsequently analyzed for translation efficiency.

Due to genetic and molecular homology between the plant- and animal-infecting members of the *Bunyaviridae* and TSWV being able to replicate in plant and insect (animal) cells, an animal T7-driven expression system was used to analyse translation of TSWV mutant/chimaeric constructs (Materials and Methods). In a follow-up study, the use of this cell system would then allow an immediate functional and comparative analysis of constructs containing the IR or hairpin structure sequence from ambisense RNA segments of the animal-infecting bunyaviruses or arenaviruses.

The first set of variants from pREN-H^A/U-rich^ differed at the 3′ termini, i.e. either lacked the entire predicted hairpin structure sequence (construct pREN-NoH, Fig. 2B), or instead contained a poly(A) tail coding sequence of 40 nucleotides (pREN-polyA, Fig. 2B). BHK-21 cells were first infected with vv-T7 and then co-transfected with the mutant REN luciferase constructs and a firefly (FF) luciferase plasmid (pIRES-FF) as internal control. While the REN luciferase gene, flanked with the 5′ and 3′ UTR of the N gene and including the sequence for the hairpin structure, showed good expression levels, no luciferase expression was observed when the hairpin structure sequence was deleted from this REN construct (Fig. 2B). When the REN sensor construct from which the hairpin was deleted, was being provided a 3′ poly(A) tail-encoding sequence (pREN-polyA), high levels of luciferase activity were recovered again (Fig. 2B). To exclude that differences in luciferase expression were not due to differences or even absence of transcription, transcriptional expression of all mutant REN constructs was verified by semi-quantitative RT-PCR and revealed similar levels (Fig. S1). Altogether, the results indicate the requirement/importance of the TSWV hairpin sequence for translation.

### Requirement of 5′ and 3′ UTR interaction for translational enhancement

**Figure 3:**
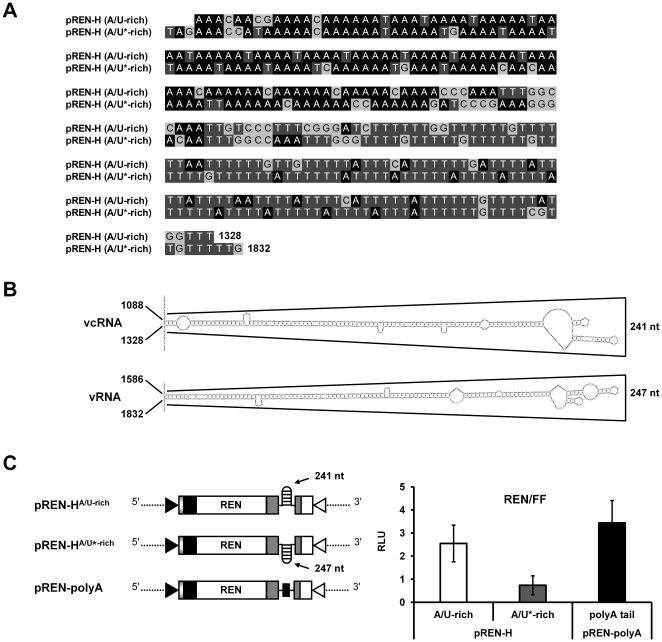
Requirement of the 3′ UTR of TSWV mRNAs in translation. (A) Sequence alignment of the TSWV N gene 3′ UTR (pREN-H^A/U-rich^) and its reverse complement (pREN-H^A/U*-rich^). (B) Mfold predictions of the highly AU-rich sequence in the viral sense RNA (vRNA) flanking the 3′ end of the NSs ORF (pREN-H^A/U*-rich^, panel A), and the analogous sequence in the viral complementary RNA (vcRNA) flanking the 3′ end of the N ORF (pREN-H^A/U-rich^, panel A). (C) Luciferase activity measured from BHK-21 cells infected with vv-T7 and subsequently co-transfected with 100 ng of expression REN constructs (pREN-H^A/U-rich^, pREN-H^A/U*-rich^, or pREN-polyA) and 0.5 ng of pIRES-FF as internal control. The relative luciferase expression (REN/FF) was measured after 23 h post transfection. Error bars show the standard deviations from the means of three replicate experiments.

In order to determine whether the observed translation of pREN-H^A/U-rich^ required the presence of a hairpin structure at the 3′ end of the mRNA or just the IR sequence, a reporter construct was produced (pREN-H^A/U*-rich^) that contained the reverse complementary copy of the IR sequence encoding the hairpin structure (Fig. 3). While this construct now contained a different sequence at its 3′ end, i.e. the sequence normally involved in transcription termination of the ambisense S RNA encoded NSs gene, it still was able to fold into a similar hairpin structure [34] (Fig. 3, A and B). The construct was transfected to vv-T7-infected BHK-21 cells and its translational activity was measured. As a control, transcriptional expression of all mutant REN sensor constructs was verified by semi-quantitative RT-PCR and revealed similar transcription levels (Fig. S1). Although both hairpin constructs, pREN-H^A/U-rich^ and pREN-H^A/U*-rich^, are highly AU-rich and share structural homology to each other (Fig. 3A), a significant decrease of luciferase activity was observed with the reverse complementary pREN-H^A/U*-rich^ construct when compared to pREN-H^A/U-rich^ (ANOVA, *P* = 0.024) (Fig. 3C).

**Figure 4:**
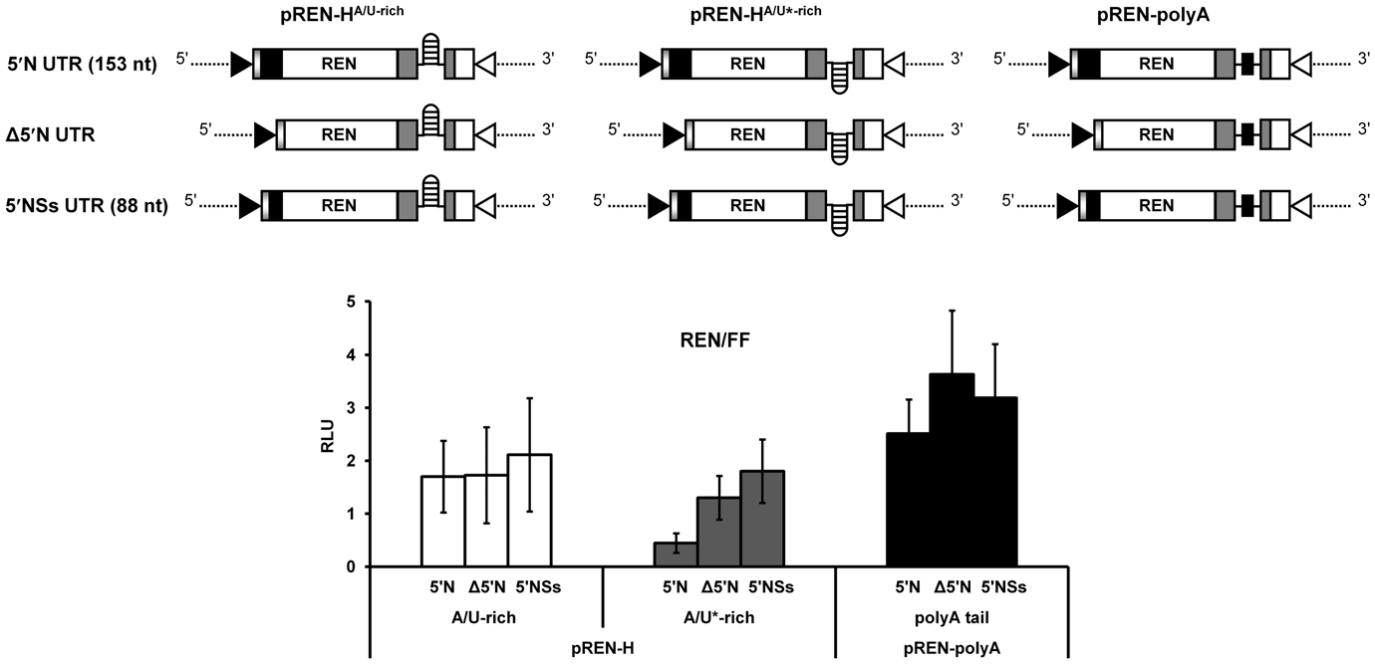
Requirement of the 5′ UTR sequence in translation. BHK-21 cells were infected with *Vaccinia virus*, and subsequently co-transfected with 100 ng of the indicated REN constructs and 0.5 ng of pIRES-FF as internal control. The relative luciferase expression (REN/FF) was measured after 23 h post transfection. Error bars show the standard deviations from the means of three replicate experiments.

To enhance the recruitment of ribosomes during translation of eukaryotic mRNAs, the 5′ and 3′ UTR sequences may also directly interact and lead to the formation of a pseudo-circularized mRNA molecule, as demonstrated for *Tomato bushy stunt virus* [35]. The reduction in luciferase activity of pREN-H^A/U*-rich^ could thus be due to the presence of heterologous 5′ and 3′ UTR sequences, i.e. the 5′ UTR originating from the TSWV N gene and the 3′ hairpin encoding IR from the TSWV NSs gene, and their inability to interact. To analyze whether the 3′ hairpin sequence acts in concert with the 5′ UTR, the latter sequence was either removed from the REN sensor hairpin constructs or replaced by the 5′ NSs UTR (Fig. 4).

Luciferase expression analysis revealed no significant differences in the activity of the N-based hairpin construct lacking the 5′ UTR or having the 5′ NSs UTR compared to pREN-H^A/U-rich^ having the homologous 5′ and 3′ UTR sequences (ANOVA, *P* = 0.971 and *P* = 0.562 respectively). However, the previous loss of reporter activity observed with the NSs-based hairpin construct harbouring the 5′ N UTR (pREN-H^A/U*-rich^), and recovery after the addition of the 5′ NSs UTR (Fig. 4) indicated that the presence of both 5′ and 3′ UTR sequences of the NSs gene was required to enhance translation. There was a statistical significance between the NSs-based hairpin construct harbouring the 5′ N UTR and this one harbouring the 5′ NSs UTR (ANOVA, *P* = 0.006). The observation that the N gene hairpin structure encoding 3′ UTR sequence could be replaced by its reverse complement, able to fold into a similar structure, pointed towards the requirement for the hairpin structure rather than a specific nucleotide sequence.

### Rescue of TSWV translation by an IR-encoding hairpin structure sequence from a distinct tospovirus

**Figure 5:**
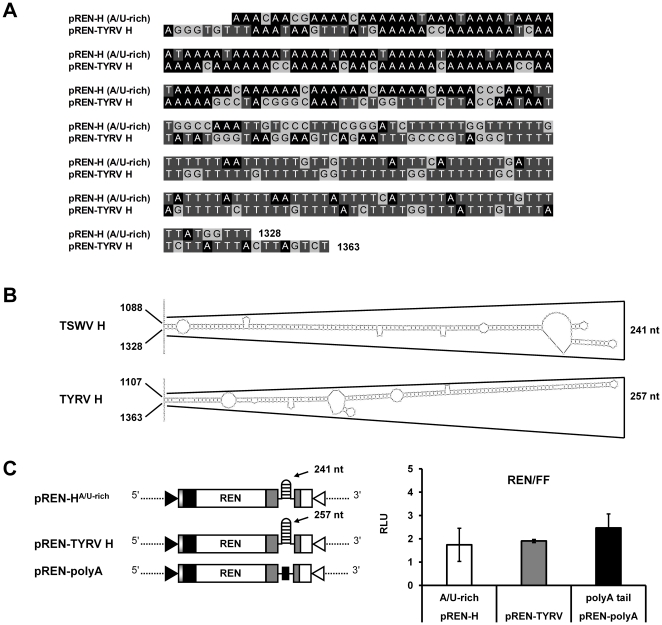
Comparison of the predicted hairpin structure sequence from TSWV (N gene transcript) with the analogous one from TYRV. (A) Alignment of the TSWV and TYRV N-based hairpin structure sequence. (B) Predicted hairpin structure at the 3′ end of the N gene of TSWV and TYRV respectively. (C) BHK-21 cells were infected with *Vaccinia virus* and transfected with 100 ng of either pREN-H^A/U-rich^, pREN-TYRV H, or pREN-polyA. In addition to the REN construct, 0.5 ng of pIRES-FF was added as internal control. After 23 h, the cells were lysed and assayed for relative luciferase activity. Error bars show the standard deviations from the means of three replicate experiments.

To further substantiate the finding that the hairpin structure, rather than a sequence specific element within the IR, is involved in translation of the TSWV S RNA-encoded N and NSs genes, the hairpin structure sequence within the TSWV N-gene 3′ UTR was exchanged for the one from a completely distinct tospovirus, i.e. *Tomato yellow ring virus* (TYRV). Whereas the TYRV S RNA encoded hairpin structure sequence from the N gene is also AU-rich, it is a bit larger in size than the one from TSWV (Fig. 5, A and B). When the TSWV hairpin structure sequence from pREN-H^A/U-rich^ was replaced by the one from TYRV (construct pREN-TYRV H), no significant difference in luciferase activity was observed in BHK21 cells between the two hairpin constructs (ANOVA, *P* = 0.711)(Fig. 5C).

### The A-rich stretch of the predicted hairpin structure suffices for translation

**Figure 6:**
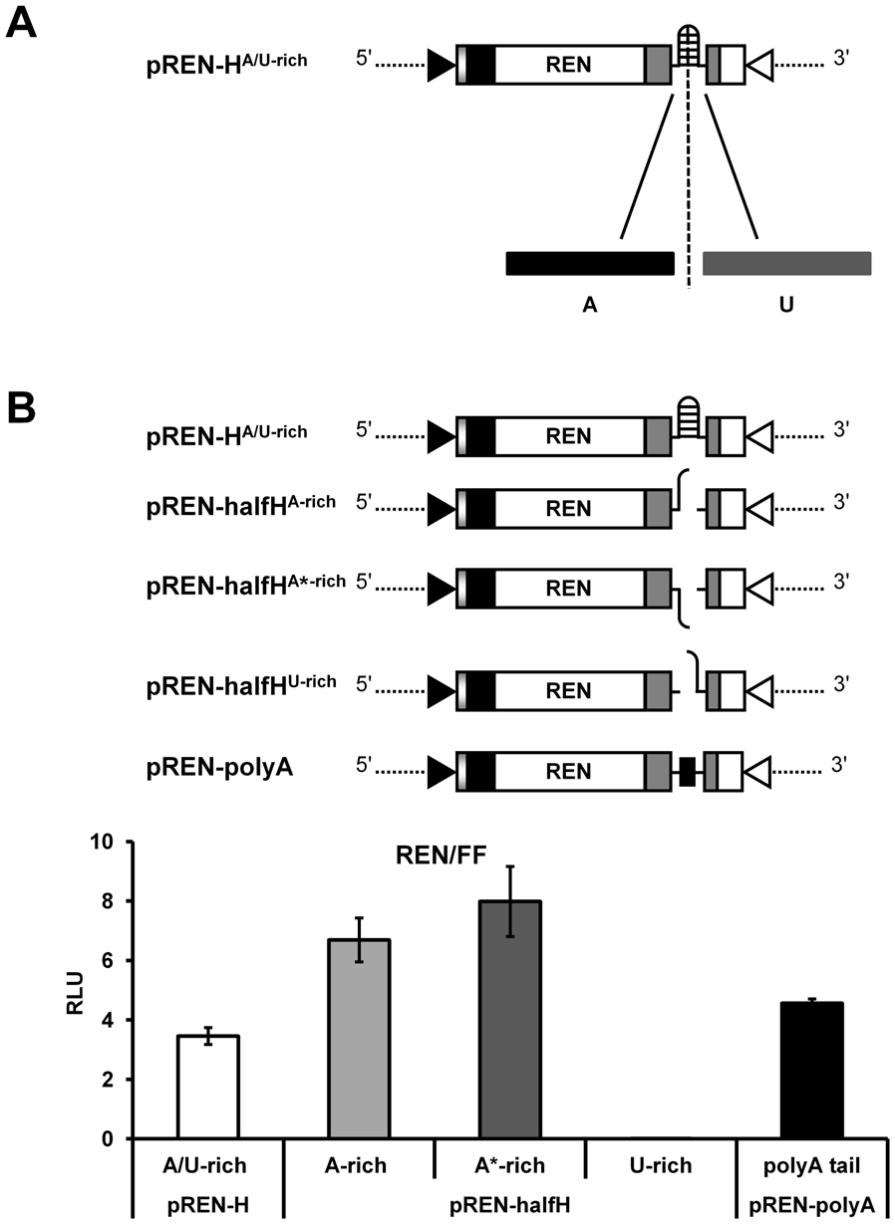
Analysis of the A- and U-rich part of the predicted hairpin structure sequence in translation. (A) Localization of the A- and U-rich part within the predicted hairpin structure sequence. (B) BHK-21 cells were infected with vv-T7 and co-transfected with 100 ng of pREN sensor constructs (pREN-H^A/U-rich^, pREN-halfH^A-rich^, pREN-halfH^A*-rich^, pREN-halfH^U-rich^, or pREN-polyA) and 0.5 ng of pIRES-FF as internal control. Relative luciferase expression was measured after 23 h post transfection. Error bars show the standard deviations from the means of three replicate experiments.

A close look at the IR showed that the first half of the predicted hairpin structure sequence contained stretches rich in A residues followed by a second half rich in U residues (Fig. 3B and 6A). For both the N and NSs gene transcripts the first part of the hairpin structure sequence (∼125 nts) contained ∼90 A residues while their similarly sized and complementary, second parts of the hairpin structure contained ∼87 U residues. Due to this sequence arrangement, it could not be excluded that only the first half of the predicted hairpin sequence mimicked a natural poly(A)-tail. To analyze this possibility, two mutants of pREN-H^A/U-rich^ were made, referred to as pREN-halfH^A-rich^ and pREN-halfH^U-rich^, from which the U- or A-rich part was lacking respectively (Fig. 6B). Furthermore, mutant pREN-halfH^A^*^-rich^ was made, containing the A-rich sequences from the reverse complementary strand of the hairpin, i.e. the A-rich sequence part of the predicted hairpin structure of the NSs gene transcript.

Analyses of these constructs on luciferase expression interestingly revealed a significant two fold increase of luciferase activity with mutants pREN-halfH^A-rich^ and pREN-halfH^A^*^-rich^ compared to the plasmid containing the entire hairpin structure sequence (ANOVA, in both cases *P*<0.001) (Fig. 6B), while no activity was measured with mutant pREN-halfH^U-rich^ (Fig. 6B). Transcriptional expression of all REN sensor constructs was verified and observed to be similar based on semi-quantitative RT-PCR (Fig. S1). These findings supported the possibility that the A-rich sequence within the hairpin structure sequence could assist in translation by mimicking a poly(A) tail.

### Requirement of viral (N and NSs) proteins in translation of hairpin structure sequence containing sensor constructs

**Figure 7:**
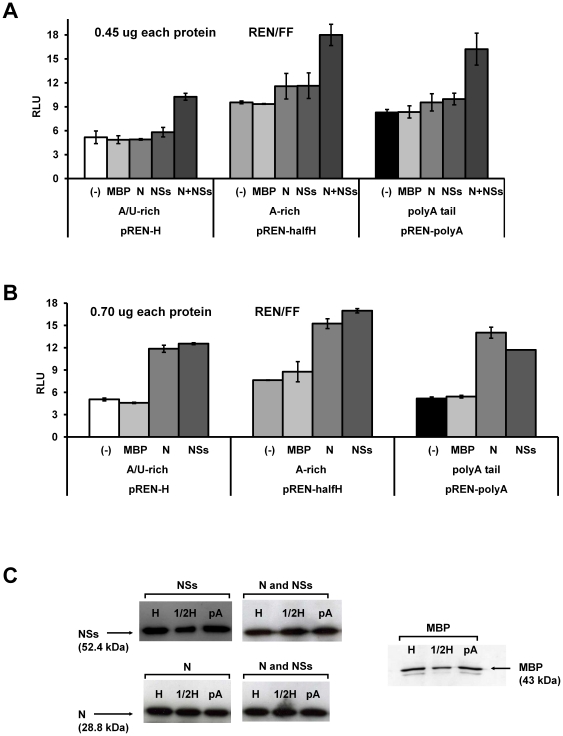
Influence of N and NSs on translation. BHK-21 cells were infected with *Vaccinia virus* and co-transfected with 100 ng of expression vectors encoding REN luciferase, FF luciferase and MBP, N, NSs, combination of N and NSs, or pUC19 at the amount of 450 ng (A) and 700 ng (B). pUC19 was added as negative control. Luciferase expression was measured 23 h post transfection. The relative luciferase expression is shown, corrected for the internal FF control (REN/FF). (C) Cells were analysed for expression of MBP, N, or NSs by Western blotting and using antisera specific for MBP, N or NSs respectively. Abbreviation: MBP, Maltose binding protein; N, nucleoprotein; NSs, non-structural protein; H, hairpin; ½H, half hairpin; pA, polyA; (-), negative control.

While previous experiments indicated an important role of the TSWV hairpin structure sequence in translation, although do not rule out a poly(A)-tail mimicking effect either, the requirement of the viral N and NSs proteins in translation was tested. To this end, the hairpin luciferase constructs pREN-H^A/U-rich^, pREN-halfH^A-rich^, and pREN-polyA were expressed in BHK21 cells in the absence or presence of expression constructs pTUG-NSs and/or pTUG-N. As a negative control, a MBP (Maltose binding protein) expression construct was included. As another negative control from which no protein would become expressed, pUC19 vector DNA was used as MOCK. Whereas no significant difference in luciferase activity was observed when the N or NSs gene constructs were added individually (450 ng each) (ANOVA, in all cases *P*>0.1), a significant increase was observed in the presence of both proteins (450 ng expression constructs each) (ANOVA, in all cases *P*<0.001) compared to the addition of the MBP (Fig. 7A). When the N and NSs gene constructs were added in a higher concentration (700 ng each), translation was even more strongly enhanced (Fig. 7B) Translation was not analysed in the presence of both N and NSs at higher concentrations, as this (together with the p-IRES-FF internal control DNA) would exceed the total amount of 1 ug of DNA that could be transfected to the cells and due to toxicity could negatively affect the expression levels. In all samples MBP, N, and NSs protein could be detected at 23 hours of post transfection (Fig. 7C).

## Discussion

The mRNAs from the majority of segmented (-) ssRNA viruses are not polyadenylated as common eukaryotic mRNAs, but instead feature translation enhancing 3′ UTRs. In general, the mechanism that allows these viral mRNAs to be efficiently translated in infected cells is poorly understood. In this study, we provide evidence that the 3′ hairpin structure sequence of TSWV ambisense S RNA-encoded mRNAs constitutes a translation enhancer that mediates efficient translation in the absence of a common poly(A) tail. Substitutions and deletions at the 3′ end, strongly suggest that the hairpin structure sequence is required for translation. Deletion of the hairpin sequence resulted in diminished translation rates, while its substitution for another slightly different hairpin encoding sequence, i.e. from its reverse complement or from another distinct tospovirus, still supported translation. The lower levels of translation in case of a REN sensor construct with a hairpin encoding sequence from the reverse complement and its recovery by complementation with the corresponding homologous 5′ UTR implies a concerted action, i.e. interaction, of the 5′ and 3′ UTR. The possibility to exchange the hairpin structure sequence among different tospoviruses indicates that these viruses share a common protein expression strategy where the hairpin structure rather than particular sequences might be the key feature in translation. Due to the strong ancestral relationship between TSWV and the animal-infecting bunyaviruses the results described here and obtained in mammalian cells, most likely apply to other ambisense encoding bunyaviruses as well. On the other hand, this study does not rule out the possibility that in plant cells some (additional) host factors might influence TSWV transcription/translation. This still remains to be investigated.

**Figure 8:**
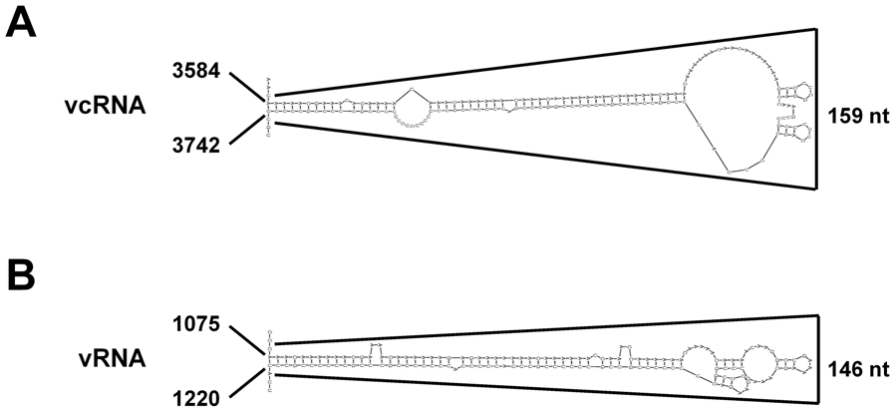
RNA folding predictions of TSWV M segment. Mfold predictions of the highly AU-rich IR in the vcRNA flanking the 3′ end of the G precursor ORF (A), and the analogous sequence in the vRNA flanking the 3′ end of the NSm ORF (B), . Abbreviation: vRNA, viral sense RNA; vcRNA, viral complementary RNA.

While the TSWV S RNA specific mRNAs are shown to contain the entire hairpin structure sequence at their 3′ end [13], stable secondary hairpin structures are predicted at the IR of both highly AU-rich ambisense S and M RNA segments (Fig. 3B and 8). It is not unlikely that these structures are also involved in mRNA transcription termination similar to what has been described for other ambisense RNA viruses like the *Tacaribe* and *Lymphocytic choriomeningitis* (LCMV) arenaviruses [9], [14], [15]. Transcription termination of LCMV mRNAs has been suggested to be reminiscent of rho-independent transcription in prokaryotes, in which termination also occurs at several positions 3′ of a GC-rich hairpin structure [36]–[38]. For the *Uukuniemi phlebovirus* transcription termination of the S RNA specific mRNAs was reported to similarly occur 3′ of an AU-rich intergenic hairpin structure [11]. On the other hand, the 3′ ends of the mRNAs of *Punta toro phlebovirus* seemed to map near to the top of a 200 base-pair predicted AU-rich hairpin structure [8].

The overall efficiency in terms of transcription and translation by the TSWV hairpin constructs raises the question of why the viral segments carry sequences that apparently can fold into structures while the transcripts can also do with the A-rich stretch only. Although a clear answer to this question has not been provided, one can speculate that such a structure provides stability of the mRNAs for exonuclease cleavage. Considering the fact that among the ambisense RNA segments from the bunyaviruses (genus *Phlebovirus* and *Tospovirus*) and arenaviruses, besides AU-rich also GC-rich hairpin folding structures are found involved in transcription termination and present in viral transcripts, strongly supports the idea that the RNA folding structure is the primary element required for translation and in which the A-rich stretches from AU-hairpin folding structures can additionally support translation in a concerted poly(A) tail binding protein (PABP)-dependent manner. For cellular mRNAs harboring a common PABP supports circularization and thereby i) stabilizes the ribonucleoprotein complex involved in translation initiation, ii) helps protect the mRNA from decay, iii) mediates ribosome recycling, and/or iv) promotes preferential translation of full-length messages [27], [28], [39], [40]. For the ambisense arenaviruses and bunyaviruses some or all of these activities may be facilitated by a hairpin folding structure, as a functional equivalence to the poly(A) tail. During such translation strategy, the hairpin structure may assists in circularization of the mRNA through specific binding of a viral protein for efficient translation, prior to the sequential recruitment of additional translation initiation factors to the 5′ end of the mRNA. Functional replacement of a poly(A) tail-PABP complex has been reported for many plant-infecting RNA viruses, e.g. *Tobacco mosaic virus* and *Alfalfa mosaic virus* [27], [28], [41]–[47]. For TSWV, such model is supported by the observation that the viral NSs and N proteins enhanced the translation of REN sensor constructs containing a 3′ hairpin structure. In this process the N and NSs proteins may likely act as a functional analog of PABP. The involvement of NSs in translation is not completely surprising, since the NSs proteins of several bunyaviruses have previously been implied to play some role in translation [48]–[52]. Since the TSWV NSs protein has recently been shown to exhibit affinity to short (si- and mi-) and long double stranded (ds)RNA [53], it is tempting to hypothesize that NSs binds to the hairpin structure to prevent its recognition and subsequent degradation in plants by RNaseIII dicer-like (DCL) proteins, while simultaneously supporting translational enhancement of viral transcripts by circularization. The involvement of the viral N protein in translational enhancement is more intriguing and so far has only been reported similarly for the Sin Nombre hantavirus N protein [54]. Although speculative, the N protein could be required in binding to the 5′ cap-structure to prevent decapping of the mRNA by decapping enzymes and thereby stabilizing viral transcripts. This hypothesis is being supported by the affinity of Sin Nombre virus N for cap-structures in cytoplasmic processing bodies (P bodies), discreet cytoplasmic foci which serve in mRNA degradation as well as cellular storage sites for mRNA [55]–[57].

The observation that only the A-rich part of the hairpin structure suffices for translation, even leaves the possibility that the AU-rich hairpin structures rely on a concerted action of viral proteins and PABP. A concerted action (interaction) would also explain why both N and NSs proteins, vice versa, stimulate translation of REN sensor constructs containing a poly(A) tail. A concerted action of viral proteins and PABP in translational enhancement is not unique as this has been earlier reported for influenza virus, where the viral NS1 protein was found to interact with PABP and also with elF4G (eukaryotic translation initiation factor) [58]–[63]. Whether PABP indeed plays an (essential) role during translation of TSWV transcripts from the ambisense S and M RNA segments remains to be analyzed, e.g. by performing translation studies in a PABP-knock down environment.

## Methods

### Viruses

Recombinant *Vaccinia virus* MVA-T7 (attenuated *Vaccinia virus* containing a copy of the T7 RNA polymerase gene) [64], [65] was used for T7 RNA polymerase driven expression of cDNA constructs. TSWV [66] was propagated in *N. benthamiana* plants. Inoculum to infect plants was obtained by grinding systemically infected plant leaves in a 0.5% Na_2_SO_3_ solution. The inoculum was applied by softly rubbing the leaves using carborundum powder.

### Construction of plasmids

To investigate the role of the TSWV hairpin structure in translation initiation, different constructs were made. TSWV constructs were generated from plasmid pTOS-S Dual [67], by using PCR amplification and cloning procedures. Briefly, this plasmid consists of a full-length DNA copy of the viral complementary strand of the ambisense S RNA cloned into a pUC19 plasmid containing a T7 promoter-terminator cassette. Luciferase marker genes were added by replacing the N and NSs genes with the REN and FF luciferase gene respectively to allow sensitive detection and quantification of translational activity.

pREN-H^A/U-rich^, pREN-NoH and pREN-polyA were PCR amplified from pTOS-S Dual by using primer TSWV S-hepδ to remove the NSs-FF ORF and primers TSWV S-H, TSWV S-NoH and TSWV S-pA respectively. Deletion of the NSs-FF ORF from the expression plasmids allowed the use of the FF luciferase pIRES-FF vector as internal control. To further mimic the authentic transcripts, all expression plasmids were re-amplified by using primers AlMV3-N-Fr and T7-AlMV3-Rv in order to add an AlMV3 RNA 3 leader immediately after the T7 promoter.

Amplified PCR fragments were separated by electrophoresis in a 1% agarose gel and purified using the GFX PCR DNA and Gel Band purification kit (GE Healthcare, Buckinghamshire, UK). Purified products were restriction enzyme digested with MSc I and re-ligated using T4 DNA ligase (Invitrogen). The nucleotide sequence of individual clones was verified by the dideoxynucleotide chain termination method using standard M13 sequencing primers and ultra-high throughput (ABI Prism 3700) DNA sequencer (Greenomics™, Wageningen University and Research Centre; The Netherlands).

A reverse complementary hairpin sequence of pREN-H^A/U-rich^, referred to as pREN-H^A/U^*^-rich^, was made as follows. pREN-H^A/U-rich^ was re-amplified with primers H-SpeI-Fr and H-NcoI-Rv. A second amplification of pREN-H^A/U-rich^ was performed with primers RlucSpeI-Fr and RlucNcoI-Rv to obtain the appropriate vector for inserting the reverse complement hairpin. Both hairpin and vector were Spe I/Nco I digested, purified and subsequently ligated together. For the TYRV hairpin construct (pREN-TYRV H), the hairpin sequence of TYRV was PCR amplified using primers TYRV-Fr and TYRV-Rv. The fragment obtained was ligated into the backbone of Spe I/Nco I digested and purified pREN-H^A/U^*^-rich^. A mutant of pREN-H^A/U-rich^ (pREN-halfH^A-rich^) from which the U-rich part of the hairpin was removed, was made by MSc I digestion of plasmid pREN-H^A/U-rich^, GFX purification and re-ligation. In a similar way, mutant pREN-halfH^A^*^-rich^ was made by Msc I digestion of pREN-H^A/U^*^-rich^. To obtain pREN-halfH^U-rich^, the U-rich fragment of the hairpin was excised from pREN-H^A/U-rich^ by Msc I digestion, and after purification subsequently ligated into Msc I digested pREN-NoH.

For analysis of the 5′ UTR, the luciferase plasmids pREN-H^A/U-rich^, pREN-H^A/U^*^-rich^, and pREN-polyA were modified. Therefore, the plasmid-specific 5′ UTR (148 nucleotides) of the N-REN ORF was removed by PCR re-amplification using primers UTRdel-Fr and UTRdel-Rv. The resulting constructs were referred to as pREN-H^A/U-rich^ Δ5′UTR, pREN-H^A/U*-rich^ Δ5′UTR, and pREN-polyA Δ5′UTR and expressed a luciferase transcript without 5′ UTR. For exchanging the 5′ N UTR with the 5′ NSs UTR, pREN-H^A/U-rich^, pREN-H^A/U^*^-rich^, and pREN-polyA were re-amplified with primer NSsUTR-Fr and NSsUTR-Rv harbouring part of the 88 nt 5′ NSs UTR. These modified REN constructs were referred to as pREN-H^A/U-rich^ 5′NSsUTR, pREN-H^A/U*-rich^ 5′NSsUTR, and pREN-polyA 5′NSsUTR.

The ORF coding for the TSWV N protein was RT-PCR amplified using primers pN-Fr and pN-Rv and cloned into the BamH I site of pTUG3, resulting in plasmid pTUG-N. The ORF for the TSWV NSs protein was cloned into pTUG3 as a BamH I fragment from pAc33DZ1/NSs [68], resulting in pTUG-NSs. Additionally, the MBP ORF was PCR amplified from a donor vector with primers MBP-Fr and MBP-Rv and cloned into pTUG3 as a BamH I fragment. Plasmid pUC19 was used as a MOCK in the experiments involving the N and NSs proteins. All constructs were checked by restriction analysis and sequencing. Primer sequences are listed in Supplementary Table S1.

### Cell culture and infection/transfection

BHK-21 cells were maintained as a monolayer in Glasgow MEM (GMEM; Gibco® Invitrogen, Breda, The Netherlands), supplemented with 1× Tryptose phosphate broth solution (Sigma-Aldrich Chemie B.V., Zwijndrecht, The Netherlands), 10% FBS, 100 U/ml·penicillin, and 100 µg/ml streptomycin. The cells were cultured in a 5% CO_2_ atmosphere at 37 C.

Cells grown in serum-free GMEM were inoculated at 37°C with *Vaccinia virus* at a multiplicity of infection (moi.) of 10. After 1 hour post infection, the medium was removed and the cells washed once with serum-free GMEM. Transfection of cells was performed with indicated amounts of plasmid DNA by using Lipofectin transfection reagent (Invitrogen, Breda, The Netherlands) according to the manufacturer's instructions. The transfection performed for 5 h at 60–70% confluence in 24-well plates.

### Protein isolation and luciferase reporter gene assays

Protein extracts were isolated using passive lysis buffer (Promega) in accordance with the manufacturer's protocol. Luciferase expression levels were determined 23 hours after transfection, using the Dual-Luciferase® Reporter Assay (Promega). Luciferase Reporter assays were carried out by co-transfecting vaccinia infected cells with plasmids containing REN luciferase and FF luciferase under the control of T7 promoter. The transfection with the FF luciferase served as control and the luciferase activity was detected using a TD 20/20 Luminometer (Turner Design) of Promega. The relative light unit (RLU) was calculated for each sample by normalization of the REN luciferase against the FF luciferase levels.

### Western blot analysis

Protein expression was characterized after plasmid transfection by Western blot analysis. Lysate prepared for the Dual-Luciferase® Reporter Assay was separated by 12% SDS/PAGE gel. After electrophoresis, proteins were semi-dry blotted to Immobilon-P transfer membranes (Millipore Corporation, Bedford, USA) using a Bio-Rad Mini-PROTEAN system. For MBP, a specific MBP-rat primary and goat alkaline phosphotase conjugated secondary antibogy were used. The protein-IgG complexes were visualized with NBT-BCIP as substrate (Roche) according to the manufacturers' procedures. For visualization of viral proteins, specific antibody for the N or NSs protein was used conjugated to alkaline phosphotase and CSPD as substrate (Roche) according to the manufacturer's procedures.

### Folding predictions

The RNA folding prediction was performed using MFold 3.5 [34]. The most energetically stable secondary structure is shown.

## Supporting Information

Figure S1 **Semi-quantitative RT-PCR analysis of mRNAs of TSWV-N (REN) constructs.** RNA was isolated (Trizol) from a similar amount of cells in all experiments and its concentration determined by nanodrop. Similar amounts of RNA were reverse transcribed using a primer specific for the 3′end of the REN gene, followed by nested PCR using internal primers to render an ∼900 bp sized REN gene product. To prevent that none of the RNAs analyzed reached a plateau during the amplification protocol, the amount of amplification cycles was limited to 30. For all pREN-constructs tested, similar amounts of RNA transcripts were observed, indicating that differences in the relative LUC activity were not caused by differences in RNA transcript levels (stability). RT-PCR was performed before or after DNase I treatment (A and B respectively). To exclude that products were resulting from REN gene amplification from the transfected DNA template, PCR amplification was performed after DNase I treatment using internal primers specific for the REN gene. As controls non-transfected, *Vaccinia virus*-infected and healthy cells were included. Size markers are shown in the first lane. M1: PstI-digested λ DNA , M2: 100 bp DNA marker.
(TIF)

Table S1 **Primer sequences.**
(DOCX)
