# Supplementary figures and images for: Analysis of the Tomato spotted wilt virus Ambisense S RNA-Encoded Hairpin Structure in Translation
Source: PLoS One. 2012 Feb 21;7(2):e31013. doi: 10.1371/journal.pone.0031013 (PMC3283609; doi:10.1371/journal.pone.0031013)

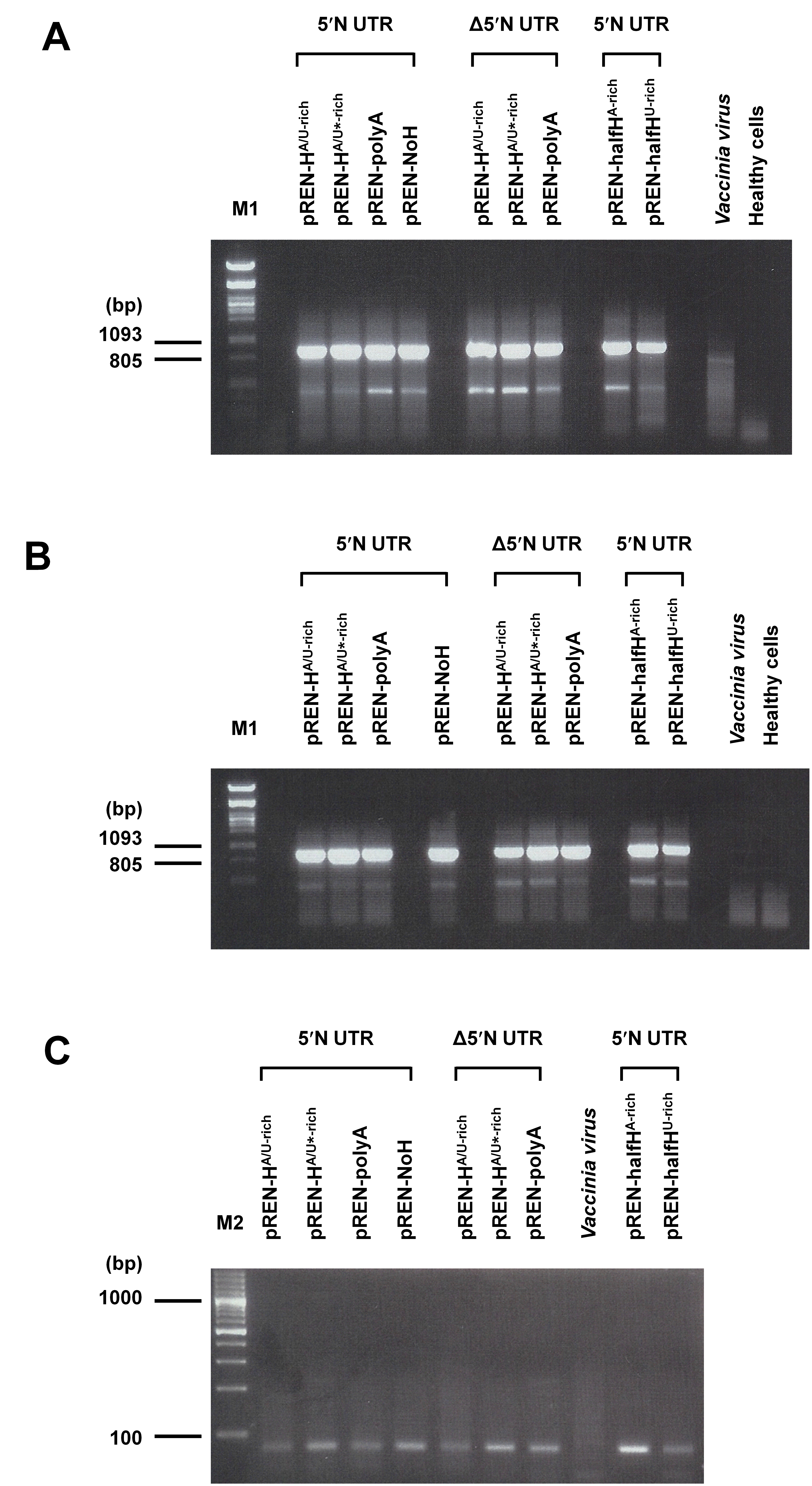

Supplement: Figure S1 — Semi-quantitative RT-PCR analysis of mRNAs of TSWV-N (REN) constructs. RNA was isolated (Trizol) from a similar amount of cells in all experiments and its concentration determined by nanodrop. Similar amounts of RNA were reverse transcribed using a primer specific for the 3′end of the REN gene, followed by nested PCR using internal primers to render an ∼900 bp sized REN gene product. To prevent that none of the RNAs analyzed reached a plateau during the amplification protocol, the amount of amplification cycles was limited to 30. For all pREN-constructs tested, similar amounts of RNA transcripts were observed, indicating that differences in the relative LUC activity were not caused by differences in RNA transcript levels (stability). RT-PCR was performed before or after DNase I treatment (A and B respectively). To exclude that products were resulting from REN gene amplification from the transfected DNA template, PCR amplification was performed after DNase I treatment using internal primers specific for the REN gene. As controls non-transfected, Vaccinia virus-infected and healthy cells were included. Size markers are shown in the first lane. M1: PstI-digested λ DNA , M2: 100 bp DNA marker. (TIF) [file pone.0031013.s001.tif]
