# Supplementary material for: Analysis of the Tomato spotted wilt virus Ambisense S RNA-Encoded Hairpin Structure in Translation
Source: PLoS One. 2012 Feb 21;7(2):e31013. doi: 10.1371/journal.pone.0031013 (PMC3283609; doi:10.1371/journal.pone.0031013)
Supplement: Table S1 — Primer sequences. (DOCX) [file pone.0031013.s002.docx]

| **Name** | **Primer sequence (5'→3')** |
| --- | --- |
| TSWV S-hepδ  TSWV S-H  TSWV S-NoH  TSWV S-pA  AlMV3-N-Fr  T7-AlMV3-Rv | CCCCTGGCCAGCTCTGGCCGGCATGGTCCC  CCCCTGGCCACTCTTTCAAATTCCTCCTGTC  CCCCTGGCCAGCACAACACACAGAAAGCAAAC  CCCCTGGCCAT(40)GCACAACACACAGAAAGCAAAC  CATTTTCAGAGCAATCGTGTCAATTTTGTGTTC  GTATTAATACTATAGTGAGTCGTATTAGGATCCCCGG |
| H-SpeI-Fr  H-NcoI-Rv  RlucSpeI-Fr  RlucNcoI-Rv  TYRV-Fr  TYRV-Rv | ACTAGTTAACAAAAACAACGAAAACAAAAAATAAATA  CCATGGTAGTAGAAACCATAAAAACAAAAAATAAAAATG  CCGGACTAGTGACAGGAGGAATTTGAAAGAG  CATGCCATGGTTTTATTATTTATTAAGCACAACACAC  CCGGACTAGTTGTAAGTCAGTAATAAAGGAGTACTAGTTTAGA  CATGCCATGGATCAGTGTGTTTAAGTTTTATCTGTCTATCA |
| UTRdel-Fr  UTRdel-Rv  NSsUTR-Fr  NSsUTR-Rv | ACGAGCTAGCCACCATGACT  CGGCTAGCGAAAATGGTATTAATACTATAGTGAGTCGT  AGAAAATCACAATACTGTAATAAGAACACAGTACCAATAACCA  GCTAGCCACCATGACTTCGA  AAGTGAGGTTTGATTATGAACAAAATTCTGACACAATTGCTCTG  AAAATGGTATTAATACTATAGTG |
| pN-Fr  pN-Rv  MBP-Fr  MBR-Rv | CCCGGATCCATGTCTAAGGTTAAGCTCACTAAGG  CCCGGATCCTCAAGCAAGTTCTGCGAGTTTTG  CGGGATCCATGAAAATCGAAGAAGGTAAACTG  CGGGATCCCTAGGATCCGAATTCTGAAATCCT |
